# Clinical Outcomes of Endoscopic Treatment for Asymptomatic and Symptomatic Common Bile Duct Stones: Incidence and Risk Factors for Post-ERCP Pancreatitis

**DOI:** 10.3390/jcm15197755

**Published:** 2026-10-08

**Authors:** Jun-Ichi Hanatani, Koh Kitagawa, Shohei Asada, Ayana Sueki, Kazuki Watanabe, Yuki Motokawa, Yui Osaki, Tomihiro Iwata, Akihiko Shibamoto, Satoshi Iwai, Yuki Tsuji, Yukihisa Fujinaga, Norihisa Nishimura, Kosuke Kaji, Shinya Sato, Tadashi Namisaki, Akira Mitoro, Hitoshi Yoshiji

**Affiliations:** 1 Department of Gastroenterology, Nara Medical University, Nara 634-8522, Japan; 2 Division of Endoscopy, Nara Medical University, Nara 634-8522, Japan

**Keywords:** choledocholithiasis, common bile duct stone, asymptomatic, endoscopic retrograde cholangiopancreatography, post-ERCP pancreatitis

## Abstract

**Background/Objectives**: Endoscopic retrograde cholangiopancreatography (ERCP) is the standard treatment for common bile duct stones (CBDSs). Current clinical guidelines recommend ERCP irrespective of symptom status. However, accumulating evidence suggests that patients with asymptomatic CBDSs are at an increased risk of post-ERCP pancreatitis (PEP). This study aimed to compare the incidence of PEP between patients with symptomatic and asymptomatic CBDSs and to identify independent risk factors for PEP. **Methods**: A total of 660 patients with a native papilla who underwent ERCP for CBDSs were retrospectively analyzed. Patients were stratified into symptomatic (*n* = 468) and asymptomatic (*n* = 192) groups. The incidence of PEP and its associated risk factors were compared between groups. **Results**: The incidence of PEP was significantly higher in the asymptomatic than symptomatic group (9.4% vs. 4.5%; *p* = 0.03). This difference was driven primarily by an increased incidence of mild PEP (6.2% vs. 2.4%; *p* = 0.02), whereas the rates of moderate and severe PEP were comparable. Pancreatic guidewire-assisted biliary cannulation was more frequent in the asymptomatic group (17.7% vs. 10.3%; *p* = 0.01) and emerged as an independent predictor of PEP. The asymptomatic group also achieved a significantly higher complete stone removal rate than the symptomatic group (99.0% vs. 95.3%; *p* = 0.02). **Conclusions**: ERCP for asymptomatic CBDSs was associated with a higher risk of PEP, primarily mild cases, but yielded favorable stone-related treatment outcomes. Therefore, ERCP is an acceptable treatment for asymptomatic CBDSs when the potential benefits and risks are thoroughly discussed with the patient beforehand.

## 1. Introduction

Choledocholithiasis is a common gastrointestinal disorder that may present with abdominal pain, back pain, nausea, vomiting, fever, and jaundice and can lead to serious complications, including acute cholangitis and acute pancreatitis [1]. Endoscopic retrograde cholangiopancreatography (ERCP) is the established first-line therapeutic modality for the management of common bile duct stones (CBDSs) worldwide [2]. Although symptomatic CBDSs are an unequivocal indication for treatment, current guidelines in Japan and other countries also recommend intervention for asymptomatic CBDSs because of the potential risk of serious complications, including acute cholangitis and biliary pancreatitis [1,3,4,5].

However, ERCP is an invasive endoscopic procedure associated with a substantial risk of procedure-related adverse events, including post-ERCP pancreatitis (PEP), bleeding, and perforation. Previous studies have reported an overall ERCP-related adverse event rate of approximately 10–15%, with procedure-related mortality occurring in fewer than 1% of cases. Among these adverse events, PEP is the most frequent complication, with a reported incidence ranging from approximately 4% to 15% [6,7,8]. Because PEP can occasionally be severe and potentially life-threatening, identifying patients at increased risk is essential for optimizing preventive strategies and improving clinical outcomes.

In recent years, evidence has been accumulating to suggest that patients with asymptomatic choledocholithiasis are at a higher risk of PEP than those with symptomatic disease [9,10,11]. However, secondary care or community hospitals have driven most available evidence in Japan, while data from university hospitals remain limited. Risk management for adverse events related to ERCP is particularly relevant at tertiary referral centers, including university hospitals, because they receive referrals for cases of CBDs that are difficult to treat at community hospitals. Furthermore, the mechanisms underlying the increased risk of PEP in patients with asymptomatic choledocholithiasis, as well as the associated risk factors, remain incompletely understood. The use of prophylactic measures against PEP, such as preprocedural rectal nonsteroidal anti-inflammatory drugs (NSAIDs), also varies considerably across studies. Consequently, it remains uncertain whether asymptomatic choledocholithiasis itself is an independent predictor of PEP or whether the increased risk is primarily attributable to procedure-related factors associated with stone extraction.

Therefore, this study aimed to compare the clinical outcomes of ERCP between patients with asymptomatic and symptomatic choledocholithiasis and to identify independent risk factors for PEP, with particular emphasis on patient characteristics and procedure-related factors.

## 2. Methods

### 2.1. Ethical Statement

This single-center, retrospective study was approved by the Nara Medical University Ethics Committee (Approval No. 3980). Written informed consent for the ERCP procedure was obtained from all patients prior to treatment. Since this was a retrospective study, the requirement for written informed consent for participation in the study was waived by the Ethics Committee, and an opt-out procedure was adopted instead.

### 2.2. Study Definitions

Asymptomatic CBDSs were defined as the absence of symptoms and laboratory abnormalities attributable to CBDSs, including elevated hepatobiliary enzyme levels and hyperbilirubinemia. The diagnosis and severity of acute cholangitis were determined according to the Tokyo Guidelines 2018 [12]. In this study, both definite and suspected diagnoses of acute cholangitis were included in the definition of cholangitis. Surgically altered anatomy was defined as a history of Billroth II or Roux-en-Y reconstruction following gastrectomy, whereas patients with Billroth I reconstruction were not classified as having surgically altered anatomy. Stone size was defined as the maximum diameter of the largest CBDS, and CBD diameter was defined as the maximum diameter of the CBD. Stones and the bile duct diameters were measured using cross-sectional imaging modalities, including computed tomography and magnetic resonance cholangiopancreatography, as well as cholangiography performed during ERCP. For each parameter, the largest value was used for analysis.

Complete stone removal was confirmed by cholangiography performed at the conclusion of the ERCP procedure. Palliative endoscopic biliary stenting (EBS) without stone removal was defined as placement of a biliary stent without an attempt at stone extraction, followed by termination of the procedure. Biliary cannulation time was defined as the interval from visualization of the major duodenal papilla to successful deep biliary cannulation during the initial ERCP session. Based on previous studies and the Japanese clinical practice guidelines, biliary cannulation time was dichotomized using a cutoff value of 10 min for the analysis of PEP risk [13,14]. Total procedure time was defined as the interval from visualization of the major duodenal papilla to completion of the ERCP procedure. For patients who required multiple ERCP sessions, the cumulative procedure time was calculated as the sum of the procedure times for all sessions. Operators were categorized as experts or trainees. Expert operators were defined as endoscopists certified as instructors by the Japan Gastroenterological Endoscopy Society and the Japan Biliary Association, with extensive experience in ERCP. All other operators were classified as trainees. ESBD was defined as endoscopic sphincterotomy (EST) followed by endoscopic papillary balloon dilation (EPBD; dilated balloon size < 12 mm), and ESLBD was defined as EST followed by endoscopic papillary large balloon dilation (EPLBD; dilated balloon size ≥12 mm).

PEP was diagnosed based on the revised Atlanta classification [15]. The diagnosis required the presence of at least two of the following three criteria: (1) abdominal pain consistent with acute pancreatitis (acute onset of persistent, severe epigastric pain often radiating to the back); (2) serum lipase or amylase activity at least three times the upper limit of normal; and (3) characteristic imaging findings of acute pancreatitis on contrast-enhanced computed tomography (CE-CT), or less commonly, magnetic resonance imaging or transabdominal ultrasonography. The severity of PEP was assessed using the criteria proposed by Cotton et al. [16]. Mild PEP was defined as clinical pancreatitis requiring hospitalization or prolongation of the planned hospital stay by 2–3 days, moderate PEP as pancreatitis requiring hospitalization for 4–10 days, and severe PEP as pancreatitis requiring hospitalization for more than 10 days.

Bleeding was defined as intraprocedural or postprocedural hemorrhage requiring endoscopic or other hemostatic intervention. Minor bleeding that resolved spontaneously without hemostatic treatment was not considered an adverse event and was excluded from the analysis. Deterioration of respiratory status was defined as a clinically significant decline in oxygenation during or after ERCP compared with the preprocedural baseline.

### 2.3. Endpoints

The primary endpoint was the incidence of PEP. The secondary endpoints included the complete stone removal rate, hospitalization days, and the incidence of other ERCP-related adverse events.

### 2.4. ERCP Procedure

ERCP procedures were performed in a dedicated endoscopy room equipped with fluoroscopy, without background noise or music. Each procedure was performed by at least two physicians, including the endoscopist and an assistant, and one nurse. Additional personnel, including radiologic technologists and medical engineers, were present as needed to provide technical support during the procedures. ERCP was performed with the patient in the prone position in most cases, whereas the left lateral or supine position was used when clinically indicated. Sedation during the procedure was primarily administered using midazolam and buprenorphine hydrochloride. Depending on the clinical condition and procedural requirement, haloperidol and/or dexmedetomidine hydrochloride were administered as adjuvant sedative agents when necessary. During the procedure, patients were continuously monitored by electrocardiography and pulse oximetry, and supplemental oxygen was administered via a nasal cannula as required. A side-viewing duodenoscope (JF-260V, TJF-260V, or TJF-Q290V; Olympus Medical, Tokyo, Japan) was used in patients with normal upper gastrointestinal anatomy or Billroth I reconstruction, whereas a balloon-assisted enteroscope (EC 450BI5 or EI-580BT; Fujifilm Medical, Tokyo, Japan) was used in patients with Billroth II or Roux-en-Y reconstruction. Selective biliary cannulation was attempted using a standard ERCP catheter (MTW ERCP Catheter; MTW Endoskopie, Wesel, Germany) with guidewire-assisted cannulation and limited contrast injection. EST was conducted using a sphincterotome (CleverCut 3V or StoneMaster V; Olympus Medical, Tokyo, Japan). Papillary balloon dilation was performed using a balloon catheter (Hurricane™ RX, CRE Wire-guided Balloon Dilator; Boston Scientific, Natick, MA, USA; REN; Kaneka Medix Corp., Osaka, Japan; or StoneMaster V; Olympus Medical, Tokyo, Japan). CBDS extraction was performed using a basket catheter (Medi-Globe 8-Wire Nitinol Basket; Medi-Globe), an extraction balloon catheter (Extraction Balloon Catheter; XEMEX, Tokyo, Japan), and/or a mechanical lithotripter (XEMEX Lithotripsy Basket Catheter; XEMEX, Tokyo, Japan), as appropriate. Straight or pigtail-type plastic stents were used for EBS and endoscopic nasobiliary drainage as indicated. No patient received preprocedural rectal NSAIDs before ERCP. Procedures performed by trainees were conducted under the supervision of expert endoscopists. When the supervising endoscopist judged that continuation of the procedure by the trainee was inappropriate or technically challenging, the procedure was taken over by an expert endoscopist. Regarding fluid management, lactated Ringer’s solution was routinely administered at approximately 100 mL/h (1500 mL in total) on the day of ERCP and at 62.5 mL/h on the following day. However, the infusion rate and duration were adjusted or discontinued according to individual patient characteristics, including age, cardiac history, and the severity of cholangitis, at the discretion of the attending physician.

### 2.5. Statistical Analysis

All Statistical analyses were conducted with EZR version 1.70 (Saitama Medical Center, Jichi Medical University, Saitama, Japan) [17], a graphical user interface for R version 4.5.2 (The R Foundation for Statistical Computing, Vienna, Austria). Continuous variables are presented as mean ± standard deviation or the median (range), as appropriate according to their distribution, whereas categorical variables are presented as frequencies and percentages. Differences between groups were assessed using Student’s *t*-test or the Mann–Whitney U test for continuous variables and the chi-square test or Fisher’s exact test for categorical variables as appropriate. All tests were two-sided, and *p*-values of less than 0.05 were considered statistically significant. To identify independent risk factors for PEP, univariable and multivariable logistic regression analyses were performed. Variables with a *p* value of less than 0.05 in the univariable analysis were entered into the multivariable logistic regression model, and odds ratios (ORs) with 95% confidence intervals (CIs) were calculated. As a sensitivity analysis, propensity score matching was performed to adjust for potential differences in baseline characteristics between the symptomatic and asymptomatic groups. Propensity scores were estimated using age, sex, performance status score, antiplatelet or anticoagulant therapy, history of cardiovascular disease, history of cerebrovascular disease, history of malignant neoplasm, dementia, Billroth I reconstruction, surgically altered anatomy, periampullary diverticulum, number of stones, stone diameter, and common bile duct diameter. A 1:1 nearest-neighbor matching method without replacement was performed using a caliper width of 0.2 standard deviations. After matching, 186 patients were included in each group.

## 3. Results

### 3.1. Study Population

**Figure 1:**
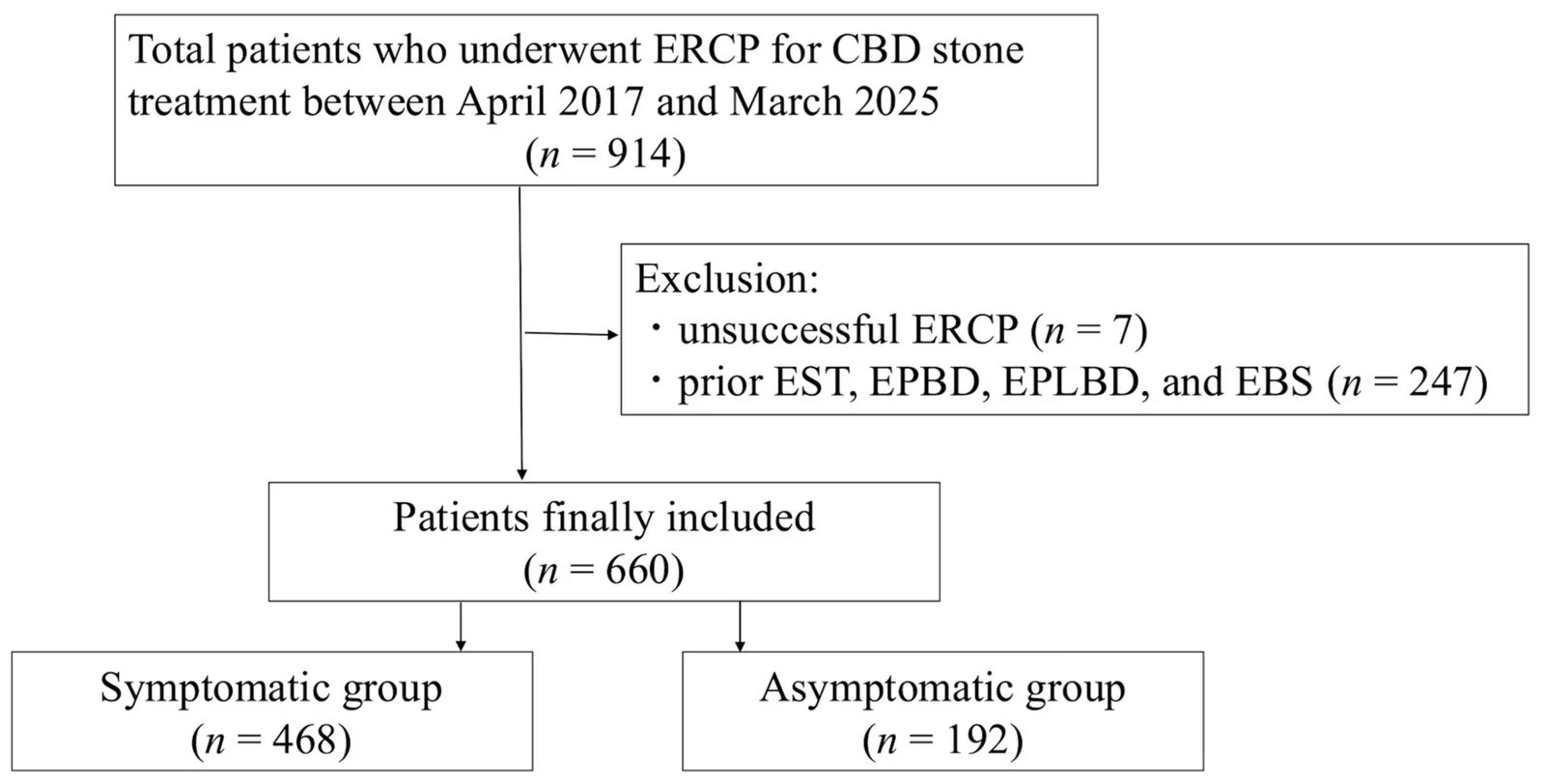
Flowchart of patient selection. Abbreviations: CBD, common bile duct; ERCP, endoscopic retrograde cholangiopancreatography; EST, endoscopic sphincterotomy; EPBD, endoscopic papillary balloon dilation; EPLBD, endoscopic papillary large balloon dilation; EBS, endoscopic biliary stenting.

The study flow chart is presented in Figure 1. Between April 2017 and March 2025, a total of 914 patients underwent ERCP for CBDSs at our institution. The diagnosis of CBDSs was established using abdominal ultrasonography, computed tomography, magnetic resonance cholangiopancreatography, or endoscopic ultrasonography. After excluding 7 patients in whom ERCP was unsuccessful and 247 patients with a prior history of EST, EPBD, EPLBD, or EBS, the remaining 660 patients with a native papilla who underwent ERCP for CBDSs were included in the final analysis. The study cohort was subsequently stratified into symptomatic (*n* = 468) and asymptomatic (*n* = 192) groups, and clinical outcomes were compared between the two groups. The data collection period was from 1 April 2026 to 31 June 2026.

### 3.2. Baseline Characteristics

Table 1 shows the baseline characteristics and ERCP procedural details of the study population. There were no significant differences between the symptomatic and asymptomatic groups in age, sex, performance status, use of antithrombotic agents, presence of periampullary diverticula, or the proportion of patients with surgically altered anatomy. Likewise, the prevalence of comorbidities was comparable between the two groups. Stone-related characteristics, including the number of stones, maximum stone diameter, and CBD diameter, were also comparable between the groups.

### 3.3. ERCP Procedural Details and Treatment Outcomes

Table 2 summarizes the ERCP procedural details and treatment outcomes. The asymptomatic group achieved a significantly higher rate of complete stone removal than the symptomatic group (99% vs. 95.3%, *p* = 0.02). Likewise, the single-session stone clearance rate was significantly higher in the asymptomatic group (88.5% vs. 48.3%, *p* < 0.01), whereas the number of ERCP sessions required for complete stone clearance was significantly lower. In contrast, palliative EBS without stone removal was performed significantly more frequently in the symptomatic group than in the asymptomatic group (3.8% vs. 0.3%, *p* = 0.02). There were no significant differences between the groups in biliary cannulation time, total procedure time, or the proportion of procedures performed by trainee endoscopists. Among the papillary interventions, EST alone was performed significantly more frequently in the asymptomatic group (56.2% vs. 47.2, *p* = 0.04), whereas EPLBD alone was performed significantly more frequently in the symptomatic group (10.7% vs. 5.2%, *p* = 0.03). No significant between-group differences were observed in the use of EPBD alone, ESBD, electrohydraulic lithotripsy, or mechanical lithotripsy. Pancreatic guidewire (PGW)-assisted cannulation was performed significantly more frequently in the asymptomatic group than in the symptomatic group (17.7% vs. 10.3%, *p* = 0.01). Pancreatic stent placement and precut sphincterotomy were performed infrequently in both groups, with no significant differences observed. Finally, the hospitalization days were significantly shorter in the asymptomatic group than in the symptomatic group (6 vs. 11 days, *p* < 0.01).

### 3.4. Incidence and Severity of Post-ERCP Pancreatitis

Table 3 summarizes the incidence and severity of PEP. The overall incidence of PEP was significantly greater in the asymptomatic group than in the symptomatic group (9.4% vs. 4.5%, *p* = 0.03). In the severity-stratified analysis, the incidence of mild PEP was also significantly higher in the asymptomatic group (6.2% vs. 2.4%, *p* = 0.04), whereas the incidence of moderate and severe PEP was comparable between the two groups. No deaths attributed to severe PEP occurred during the study period.

### 3.5. Risk Factors for Post-ERCP Pancreatitis

Table 4 summarizes the results of the univariate and multivariate analyses of risk factors for PEP. In the univariate analysis, among baseline patient characteristics excluding procedure-related variables, only asymptomatic CBDSs were significantly associated with an increased risk of PEP (odds ratio [OR], 2.20; 95% confidence interval [CI], 1.15–4.23; *p* = 0.02). Age, sex, performance status, comorbidities, and anatomical factors, including surgically altered anatomy and periampullary diverticula, were not significantly associated with PEP. Likewise, stone- and CBD-related factors were not identified as significant predictors. Among procedure-related factors, biliary cannulation time ≥10 min (OR, 3.78; 95% CI, 1.85–7.73; *p* < 0.01), EPBD alone (OR, 2.47; 95% CI, 1.16–5.28; *p* = 0.02), and PGW-assisted cannulation (OR, 5.17; 95% CI, 2.58–10.30; *p* < 0.01) were significantly associated with an increased risk of PEP. In contrast, complete stone removal, single-stage stone removal, the number of ERCP sessions, palliative EBS without stone removal, and procedures performed by trainee endoscopists were not significantly associated with PEP. Similarly, other papillary interventions and the use of mechanical lithotripsy were not identified as significant risk factors.

In the multivariate analysis, biliary cannulation time ≥ 10 min (OR, 2.36; 95% CI, 1.08–5.19; *p* = 0.03), EPBD alone (OR, 2.44; 95% CI, 1.11–5.38; *p* = 0.03), and PGW-assisted cannulation (OR, 3.46; 95% CI, 1.60–7.47; *p* <0.01) remained independent risk factors for PEP. Although asymptomatic CBDSs were significantly associated with PEP in the univariate analysis, this association was no longer statistically significant after multivariable adjustment (OR, 1.91; 95% CI, 0.98–3.75; *p* = 0.06).

### 3.6. Adverse Events Other than PEP

Table 5 summarizes the incidence of adverse events other than PEP. The incidences of bleeding, perforation, bile leakage, cholangitis, cholecystitis, respiratory deterioration, and aspiration pneumonia, and no significant differences were observed between the symptomatic and asymptomatic groups regarding other procedure-related adverse events.

### 3.7. Propensity Score-Matched Analysis

Table 6 summarizes the baseline characteristics, treatment outcomes, and PEP outcomes in the propensity score-matched symptomatic group (n = 186) and asymptomatic group (n = 186). The asymptomatic group had a significantly higher rate of single-stage stone removal, fewer ERCP sessions, and a shorter hospital stay than the symptomatic group. No significant differences were observed in the other treatment outcomes between the two groups. The incidence of PEP was significantly higher in the asymptomatic group than in the symptomatic group. When stratified by severity, mild PEP was significantly more frequent in the asymptomatic group, whereas the incidences of moderate and severe PEP were comparable between the two groups.

## 4. Discussion

Several previous studies have reported that asymptomatic choledocholithiasis is associated with an increased risk of PEP [9,10,11]. Although several studies conducted outside Japan have originated from tertiary referral or university hospitals [18,19], most Japanese studies have been conducted at community or secondary care hospitals, where the majority of ERCP procedures for choledocholithiasis are undertaken [9,11]. Accordingly, evidence from Japanese tertiary referral university hospitals remains limited. Furthermore, the use of prophylactic rectal NSAIDs varied across Japanese studies, and its potential influence on the reported incidence of PEP cannot be excluded. The present study helps address these gaps by providing clinical data from a Japanese university hospital, where patients with more complex clinical characteristics are frequently managed. In addition, because prophylactic rectal NSAIDs were not administered to any patient in this cohort, the association between asymptomatic choledocholithiasis and clinical outcomes could be evaluated without the potential confounding effect of this prophylactic intervention. In our institution, rectal NSAIDs were not routinely used for PEP prophylaxis during the study period. This was partly because only 50 mg formulations are available in Japan, whereas most evidence supporting their prophylactic efficacy is based on a 100 mg dose. In addition, prophylactic use of rectal NSAIDs is not covered by the Japanese national health insurance system [14]. Furthermore, as a university hospital, our institution manages many patients with comorbidities that may limit NSAID use.

In the present study, the incidence of PEP was significantly higher in the asymptomatic group than in the symptomatic group, despite the absence of significant differences in baseline patient characteristics between the two groups. Consistent with these findings, the propensity score-matched analysis yielded similar results. Several mechanisms have been proposed to explain the increased risk of PEP in patients with asymptomatic choledocholithiasis. First, because asymptomatic stones are not typically associated with biliary obstruction, these patients are less likely to have a dilated bile duct and more likely to have normal serum bilirubin levels, the latter of which has been identified as a risk factor for PEP [20,21]. Second, the absence of biliary obstruction may lead to a smaller papillary orifice and lower distal CBD pressure, potentially increasing the technical difficulty of selective biliary cannulation [10]. Third, some investigators have proposed that asymptomatic choledocholithiasis itself may represent an independent risk factor for PEP, irrespective of biliary anatomical characteristics or procedure-related factors [9,11].

In this study, no significant difference in CBD diameter was observed between the asymptomatic and symptomatic groups, and a nondilated CBD was not associated with an increased risk of PEP in the univariable analysis. In contrast, PGW-assisted biliary cannulation was required significantly more frequently in the asymptomatic group and remained an independent risk factor for PEP in both the univariable and multivariable analyses. Although a nondilated CBD has previously been reported to be associated with difficult biliary cannulation [22], our findings suggest that the absence of bile duct dilatation alone does not fully explain either the increased technical difficulty of biliary cannulation or the higher incidence of PEP. Rather, patients with asymptomatic choledocholithiasis may have biliary anatomical or physiological characteristics that make selective biliary cannulation more technically challenging, thereby increasing the need for PGW-assisted biliary cannulation. The association between difficult biliary cannulation, including pancreatic guidewire-assisted cannulation, and an increased risk of PEP has been well established in previous studies [23,24,25], and our findings are consistent with these observations. In the present study, EPBD and prolonged biliary cannulation time were also identified as independent risk factors for PEP, consistent with previous reports [26,27]. However, neither the frequency of EPBD nor the biliary cannulation time differed significantly between the asymptomatic and symptomatic groups. In contrast, PGW-assisted biliary cannulation was performed significantly more frequently in the asymptomatic group and remained an independent predictor of PEP in the multivariate analysis. These findings suggest that PGW-assisted biliary cannulation may play a more important role in the increased risk of PEP among patients with asymptomatic choledocholithiasis.

However, our multivariable analysis did not identify asymptomatic choledocholithiasis as an independent risk factor for PEP, in contrast to the findings of several previous studies [9,10,11]. Instead, our findings suggest that procedure-related factors, particularly the need for PGW-assisted biliary cannulation, may contribute more substantially to the increased risk of PEP in this patient population. Nevertheless, the mechanisms underlying the observed association between asymptomatic choledocholithiasis and PEP remain incompletely understood. Further large-scale prospective multicenter studies are warranted to further elucidate the underlying mechanisms and to clarify the relative contributions of patient- and procedure-related factors to the development of PEP.

Although the incidence of PEP was higher in patients with asymptomatic choledocholithiasis, whether this finding alone should preclude endoscopic treatment remains controversial. In the present study, the increased incidence of PEP was attributable primarily to mild cases, whereas the rates of moderate and severe PEP did not differ significantly between the asymptomatic and symptomatic groups. Similar findings were also observed in the propensity score-matched analysis. Moreover, the incidence of adverse events other than PEP did not differ significantly between the two groups. Notably, patients with asymptomatic choledocholithiasis achieved more favorable treatment outcomes, including a higher complete stone removal rate, fewer ERCP sessions, and a shorter length of hospital stay. In addition, the risk of PEP may be further mitigated by the routine implementation of evidence-based prophylactic measures, such as rectal NSAIDs and prophylactic pancreatic stent placement in patients requiring PGW-assisted biliary cannulation. Therefore, treatment decisions for asymptomatic choledocholithiasis should not be based solely on the increased risk of PEP. Rather, the potential benefits and risks of endoscopic intervention should be carefully balanced and discussed with patients to facilitate informed shared decision-making.

The present study has several limitations. First, this study was a retrospective, single-center study performed at a university hospital providing tertiary care, which may have introduced selection bias and limited the external validity and generalizability of the findings. Second, the selection of papillary intervention, biliary cannulation technique, and endoscopic devices was not standardized but was dependent on the discretion of the endoscopist performing the procedure. Consequently, procedure-related outcomes may have been influenced by operator-dependent factors and other unmeasured procedural characteristics. Third, operator experience may not have been uniform. Although operators were classified as experts or trainees according to whether they held instructor certification from the Japan Gastroenterological Endoscopy Society and the Japan Biliary Association, the level of ERCP experience varied among trainees. In addition, other operator-related factors, such as operator sex and the specific number of ERCP procedures performed by each operator, were not accurately available in this retrospective study. Future large-scale, preferably prospective, multicenter studies are warranted to validate our findings and to further investigate factors associated with PEP.

## 5. Conclusions

The incidence of PEP in patients with asymptomatic choledocholithiasis was higher than that in patients with symptomatic choledocholithiasis and may be associated with the more frequent need for PGW-assisted biliary cannulation. However, the increase in PEP was primarily attributable to mild cases. Furthermore, stone-related treatment outcomes were more favorable in the asymptomatic group. Therefore, ERCP for asymptomatic CBDSs is acceptable, provided that the potential benefits and risks of the procedure are thoroughly discussed with the patient beforehand.

## Figures and Tables

**Table 1:** Baseline characteristics of the study population.

	Symptomatic Group (*n* = 468)	Asymptomatic Group (*n* = 192)	*p* Value
Age, median (range), years	77 (19–100)	75 (40–98)	0.08
Sex, male/female, *n*	303/165	118/74	0.42
Performance status score ≥2, *n* (%)	33 (7.1%)	8 (4.2%)	0.21
Antiplatelet or anticoagulant therapy, *n* (%)	126 (26.9%)	57 (29.7%)	0.50
History of cardiovascular disease, *n* (%)	139 (29.7%)	52 (27.1%)	0.57
History of cerebrovascular disease, *n* (%)	54 (11.5%)	30 (15.6%)	0.16
History of malignant neoplasm, *n* (%)	136 (29.1%)	59 (30.7%)	0.71
Dementia, *n* (%)	31 (6.6%)	9 (4.7%)	0.47
Billroth I reconstruction, *n* (%)	10 (2.1%)	8 (4.2%)	0.19
Surgically altered anatomy, *n* (%)	55 (11.8%)	21 (10.9%)	0.89
Periampullary diverticulum, *n* (%)	140 (29.9%)	55 (28.6%)	0.78
Severity of cholangitis, mild/moderate/severe, *n*	300/130/26		
Number of stones, median (range), *n*	1 (1–15)	1 (1–8)	0.64
Stone diameter, median (range), mm	6 (1–32)	6 (1–18)	0.22
Common bile duct diameter, median (range), mm	10 (4–25)	9 (3–25)	0.48

**Table 2:** Endoscopic retrograde cholangiopancreatography (ERCP) procedural features and stone-related treatment outcomes.

	Symptomatic Group (*n* = 468)	Asymptomatic Group (*n* = 192)	*p*
Complete stone removal, *n* (%)	446 (95.3%)	190 (99.0%)	0.02
Single-stage stone removal, *n* (%)	226 (48.3%)	170 (88.5%)	<0.01
Palliative EBS without stone removal, *n* (%)	18 (3.8%)	1 (0.5%)	0.02
Number of sessions, median (range), *n*	1 (1–4)	1 (1–3)	<0.01
Biliary cannulation time, median (range), min	7 (1–150)	7 (1–123)	0.57
Biliary cannulation time ≥10 min, *n* (%)	190 (40.6%)	88 (45.8%)	0.23
Total procedure time, median(range), min	35 (4–256)	33 (9–176)	0.30
Total procedure time ≥30 min, *n* (%)	294 (62.8%)	113 (58.9%)	0.38
Trainee endoscopist, *n* (%)	392 (83.8%)	149 (77.6%)	0.07
EST alone, *n* (%)	221 (47.2%)	108 (56.2%)	0.04
EPBD alone, *n* (%)	54 (11.5%)	32 (16.7%)	0.10
EPLBD alone, *n* (%)	50 (10.7%)	10 (5.2%)	0.03
ESBD, *n* (%)	70 (15.0%)	24 (12.5%)	0.46
ESLBD, *n* (%)	48 (10.3%)	11 (5.7%)	0.07
Mechanical lithotripsy, *n* (%)	117 (25.0%)	35 (18.2%)	0.07
EHL, *n* (%)	5 (1.1%)	1 (0.5%)	0.68
Pancreatic guidewire-assisted cannulation, *n* (%)	48 (10.3%)	34 (17.7%)	0.01
Pancreatic stent placement, *n* (%)	5 (1.1%)	5 (2.6%)	0.16
Precut sphincterotomy, *n* (%)	6 (1.3%)	3 (1.6%)	0.72
Hospitalization days, median (range), days	11 (2–138)	6 (3–56)	<0.01

Abbreviations: EBS, endoscopic biliary stenting; EST, endoscopic sphincterotomy; EPBD, endoscopic papillary balloon dilation; EPLBD, endoscopic papillary large balloon dilation; ESBD, endoscopic papillary balloon dilation with endoscopic sphincterotomy; ESLBD, endoscopic papillary large balloon dilation with endoscopic sphincterotomy; EHL, electrohydraulic lithotripsy.

**Table 3:** Incidence and severity of post-ERCP pancreatitis.

Outcome	Symptomatic Group (*n* = 468)	Asymptomatic Group (*n* = 192)	*p*
Post-ERCP pancreatitis, *n* (%)	21 (4.5%)	18 (9.4%)	0.03
Severity, *n* (%)			
Mild	11 (2.4%)	12 (6.2%)	0.02
Moderate	6 (1.3%)	2 (1.0%)	1.00
Severe	4 (0.9%)	4 (2.1%)	0.24

**Table 4:** Univariable and multivariable analyses of risk factors for post-ERCP pancreatitis.

	Univariable Analysis			Multivariable Analysis		
	OR	95% CI	*p*	OR	95% CI	*p*
Age < 70 years	1.26	0.62–2.54	0.52			
Female	1.73	0.91–3.31	0.10			
Performance status score ≥2	0.38	0.05–2.86	0.35			
Antiplatelet or anticoagulant therapy	0.66	0.30–1.46	0.30			
History of cardiovascular disease	0.84	0.40–1.76	0.64			
History of cerebrovascular disease	0.56	0.17–1.85	0.34			
History of malignant neoplasm	1.06	0.53–2.15	0.86			
Dementia	1.32	0.39–4.47	0.66			
Billroth I reconstruction	0.94	0.12–7.21	0.95			
Surgically altered anatomy	0.87	0.30–2.52	0.80			
Asymptomatic stones	2.20	1.15–4.23	0.02	1.91	0.98–3.75	0.06
Number of stones ≥2	0.61	0.31–1.19	0.15			
Stone size ≥10 mm	0.69	0.31–1.52	0.35			
Nondilated CBD (<8 mm)	1.86	0.91–3.77	0.09			
Complete stone removal	0.68	0.15–3.00	0.61			
Single-stage stone removal	1.75	0.86–3.58	0.13			
Palliative EBS without stone removal	1.92	0.43–8.63	0.40			
Number of sessions ≥2 times	0.52	0.24–1.12	0.10			
Biliary cannulation time ≥10 min	3.78	1.85–7.73	<0.01	2.36	1.08–5.19	0.03
Total procedure time ≥30 min	1.86	0.89–3.89	0.10			
Trainee endoscopist	0.62	0.29–1.30	0.21			
EST alone	1.06	0.56–2.03	0.85			
EPBD alone	2.47	1.16–5.28	0.02	2.44	1.11–5.38	0.03
EPLBD alone	0.83	0.25–2.76	0.75			
ESBD	0.67	0.23–1.94	0.47			
ESLBD	0.00	0–Inf	0.99			
Mechanical lithotripsy	0.37	0.13–1.04	0.06			
Pancreatic guidewire-assisted cannulation	5.17	2.58–10.30	<0.01	3.46	1.60–7.47	<0.01
Pancreatic stent placement	0.00	0–Inf	0.99			
Precut sphincterotomy	0.00	0–Inf	0.99			

**Table 5:** Incidence of adverse events other than post-ERCP pancreatitis (PEP).

	Symptomatic Group (*n* = 468)	Asymptomatic Group (*n* = 192)	*p*
Bleeding, *n* (%)	32 (6.8%)	13 (6.8%)	1.00
Perforation, *n* (%)	3 (0.6%)	1 (0.5%)	1.00
Bile leakage, *n* (%)	2 (0.4%)	0 (0%)	1.00
Cholecystitis, *n* (%)	6 (1.3%)	0 (0%)	0.19
Cholangitis, *n* (%)	2 (0.4%)	4 (2.1%)	0.06
Deterioration of respiratory condition, *n* (%)	5 (1.1%)	2 (1.0%)	1.00
Aspiration pneumonia, *n* (%)	3 (0.6%)	2 (1.0%)	0.63
Other, *n* (%)	4 (0.9%)	3 (1.6%)	0.42

**Table 6:** Comparison of Baseline Characteristics, Treatment Outcomes, and Post-ERCP pancreatitis (PEP) after Propensity Score Matching.

	Symptomatic Group (*n* = 186)	Asymptomatic Group (*n* = 186)	*p*
***Baseline Characteristics***			
Age, median (range), years	77 (19–100)	75 (40–98)	0.07
Sex, male/female, *n*	112/74	113/73	1
Performance status score ≥2, *n* (%)	7 (3.8%)	8 (4.3%)	1
Antiplatelet or anticoagulant therapy, *n* (%)	45 (24.2%)	56 (30.1%)	0.24
History of cardiovascular disease, *n* (%)	48 (25.8%)	51 (27.4%)	0.81
History of cerebrovascular disease, *n* (%)	25 (13.4%)	27 (14.5%)	0.88
History of malignant neoplasm, *n* (%)	58 (31.2%)	56 (30.1%)	0.91
Dementia, *n* (%)	8 (4.3%)	9 (4.8%)	1
Billroth I reconstruction, *n* (%)	5 (2.7%)	5 (2.7%)	1
Surgically altered anatomy, *n* (%)	14 (7.5%)	21 (11.3%)	0.29
Periampullary diverticulum, *n* (%)	52 (28.0%)	54 (29.0%)	0.91
Severity of cholangitis, mild/moderate/severe, *n*	126/50/6		
Number of stones, median (range), *n*	1 (1–10)	1 (1–8)	0.78
Stone diameter, median (range), mm	5 (1–20)	6 (1–18)	0.42
Common bile duct diameter, median (range), mm	10 (5–20)	9 (3–25)	0.42
***Treatment Outcomes***			
Complete stone removal, *n* (%)	177 (95.2%)	184 (98.9%)	0.06
Single-stage stone removal, *n* (%)	91 (48.9%)	165 (88.7%)	<0.01
Palliative EBS without stone removal, *n* (%)	8 (4.3%)	1 (0.5%)	0.04
Number of sessions, median (range), *n*	1 (1–3)	1 (1–3)	<0.01
Biliary cannulation time, median (range), min	6 (1–120)	7 (1–123)	0.29
Biliary cannulation time ≥10 min, *n* (%)	70 (37.6%)	85 (45.7%)	0.14
Total procedure time, median(range), min	32 (4–189)	33 (9–176)	0.63
Total procedure time ≥30 min, *n* (%)	109 (58.6%)	110 (59.1%)	1
Trainee endoscopist, *n* (%)	157 (84.4%)	146 (78.5%)	0.18
EST alone, *n* (%)	95 (51.1%)	108 (58.1%)	0.21
EPBD alone, *n* (%)	19 (10.2%)	31 (16.7%)	0.09
EPLBD alone, *n* (%)	16 (8.6%)	9 (4.8%)	0.21
ESBD, *n* (%)	31 (16.7%)	23 (12.4%)	0.30
ESLBD, *n* (%)	15 (8.1%)	11 (5.9%)	0.54
Mechanical lithotripsy, *n* (%)	43 (23.1%)	35 (18.8%)	0.37
EHL, *n* (%)	1 (0.5%)	1 (0.5%)	1
Pancreatic guidewire-assisted cannulation, *n* (%)	24 (12.9%)	34 (18.3%)	0.20
Pancreatic stent placement, *n* (%)	2 (1.1%)	5 (2.7%)	0.45
Precut sphincterotomy, *n* (%)	3 (1.6%)	3 (1.6%)	1
Hospitalization days, median (range), days	11 (2–136)	6 (3–56)	<0.01
***Incidence and severity of PEP***			
Post-ERCP pancreatitis, *n* (%)	5 (2.7%)	18 (9.7%)	<0.01
Severity, *n* (%)			
Mild	2 (1.1%)	12 (6.5%)	0.01
Moderate	0 (0.0%)	2 (1.1%)	0.50
Severe	3 (1.6%)	4 (2.2%)	1

## Data Availability

The datasets used and/or analyzed during the current study are available from the corresponding author upon reasonable request.

## Abbreviations

The following abbreviations are used in this manuscript:

CBDS	Common Bile Duct Stones
CT	Computed Tomography
EBS	Endoscopic Biliary Stenting
EPBD	Endoscopic Papillary Balloon Dilation
EPLBD	Endoscopic Papillary Large Balloon Dilation
PEP	Post-ERCP Pancreatitis
